# Supplementary material for: Microscopic Analysis of Heterochromatin, Euchromatin and Cohesin in Cancer Cell Models and under Anti-Cancer Treatment
Source: Curr Issues Mol Biol. 2023 Oct 9;45(10):8152–72. doi: 10.3390/cimb45100515 (PMC10605351; doi:10.3390/cimb45100515)
Supplement: Supplementary file 1 [file cimb-45-00515-s001.zip › cimb-2622498-supplementary.pdf]

Supplement Materials

(A) Application of the DAPI mask

For quantitative evaluations, the cell nuclei were segmented according to the masks obtained by their individual DAPI counterstaining. This staining was usually congruent with the H3K4me3 labelling (mostly representing euchromatin). In the cases of sometimes weak H3K9me3 or cohesion labelling, the strong DAPI signal was used to separate signals inside and outside the cell nucleus. Typical Examples are shown in Figure S1.

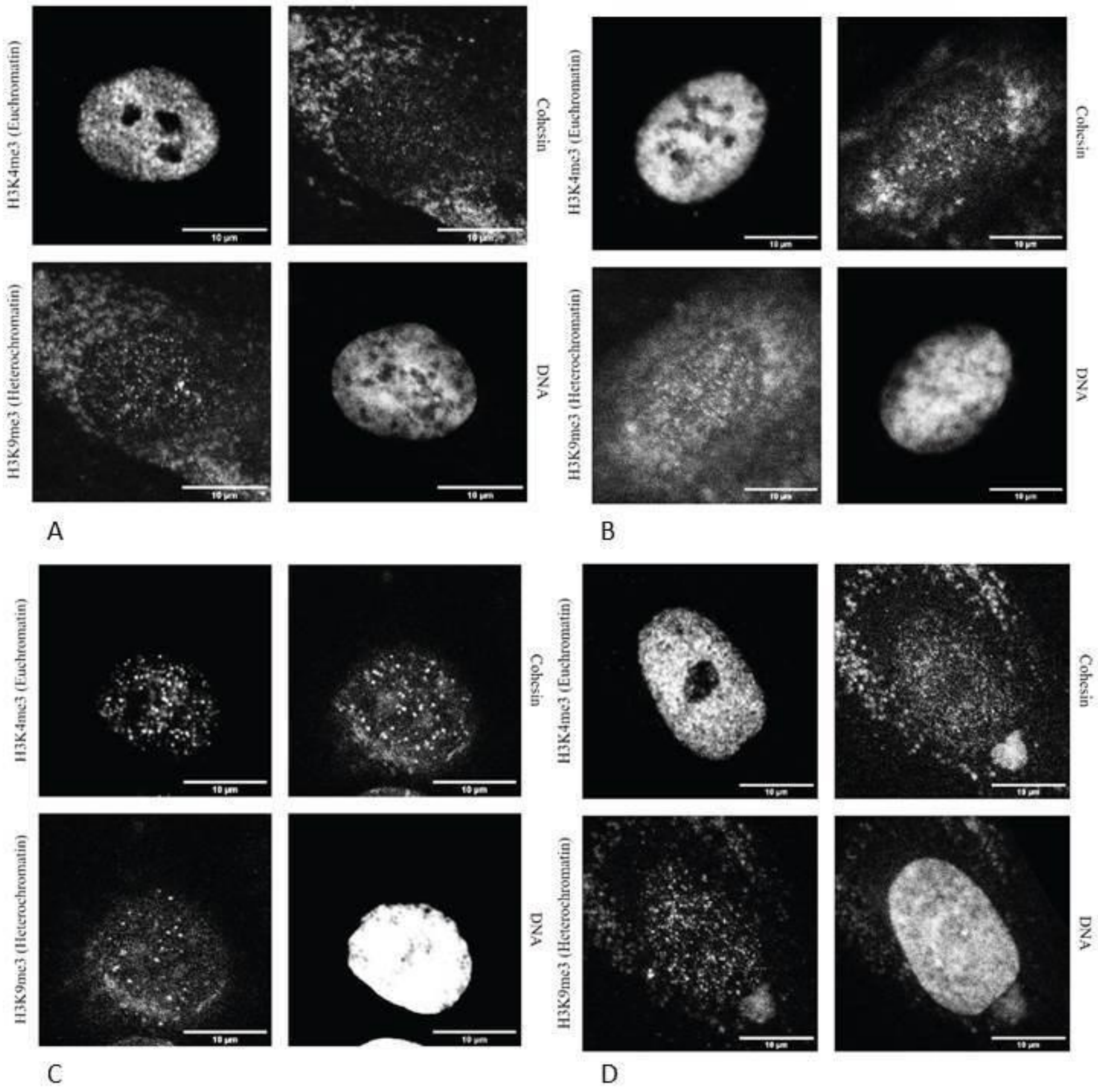

Figure S1: Representative confocal images of cell nuclei showing the application of the DAPI (=DNA) mask for fibroblasts (**A**) CRL2522dorm, (**B**) CRL2522_2Gy,1.5h; for breast cancer cells (**C**) SKBR3_4Gy,1.5h; for mouse cells (**D**) P1 Aza-. The Images show the good congruence Between the H3K4me3 (mostly representing euchromatin) and the DAPI (DNA) labelling. In case of the thin labelling of H3K9me3 (mostly representing heterochromatin) and cohesin the contrast indicates the nucleus border which is congruent to the DAPI (DNA) image. Scale bars: 10 µm.

(B) Statistical tests

For statistical comparison of the cell lines and the treatment conditions, the two-sample t-test was used for normally distributed data. In other cases the Kruskal-Wallis ANOVA test was used. In the following the tables of the p-values are shown: p-value of $p \geq 0.05$ corresponds to "non-significant" (n.s.), $p < 0.05$ to "significant" (*), $p < 0.01$ to "highly significant" (**) and $p < 0.001$ to "very highly significant" (***).

Table S1: *p*-values (*t*-test) for comparison of the size of the nuclei

Comparison	*p*-Value	Significance
CRL2522run - U87	<0.001	***
CRL2522run - SKBR3_0Gy	<0.001	***
CRL2522run - P19Aza- .	0.970	n.s.
U87 - SKBR3_0Gy	0.043	*
U87 - P19 Aza-	<0.001	***
SKBR3_0Gy - P19 Aza-	<0.001	***
CRL2522run - CRL2522dorm	0.007	**
CRL2522run - CRL2522wake	0.086	n.s.
CRL2522dorm - CRL2522wake	0.203	n.s.
CRL2522run - CRL2522_2Gy,1.5h	0.139	n.s.
CRL2522run - CRL2522_4Gy,1.5h .	0.057	n.s.
CRL2522_2Gy,1.5h - CRL2522_4Gy,1.5h	0.003	**
CRL2522_run - CRL2522_2Gy,8h	0.428	n.s.
CRL2522_2Gy,1.5h - CRL2522_2Gy,8h	0.439	n.s.
SKBR3_0Gy - SKBR3_4Gy,1.5h	0.027	*
P19Aza- - P19Aza+	0.790	n.s.
SKBR3_4Gy,1.5h - CRL2522_4Gy,1.5h	<0.001	***

Table S2: *p*-values (ANOVA-test) for comparison of the amount of euchromatin of the nuclei

Comparison	*p*-Value	Significance
CRL2522run - U87	0.907	n.s.
CRL2522run - SKBR3_0Gy	<0.001	***
CRL2522run - P19Aza- .	0.129	n.s.
U87 - SKBR3_0Gy	<0.001	***
U87 - P19 Aza-	<0.001	***
SKBR3_0Gy - P19 Aza-	<0.001	***
CRL2522run - CRL2522dorm	<0.001	***
CRL2522run - CRL2522wake	0.393	n.s.
CRL2522dorm - CRL2522wake	<0.001	***
CRL2522run - CRL2522_2Gy,1.5h	0.044	*
CRL2522run - CRL2522_4Gy,1.5h .	<0.001	***
CRL2522_2Gy,1.5h - CRL2522_4Gy,1.5h	<0.001	***
CRL2522_run - CRL2522_2Gy,8h	0.099	n.s.
CRL2522_2Gy,1.5h - CRL2522_2Gy,8h	0.585	n.s.
SKBR3_0Gy - SKBR3_4Gy,1.5h	<0.001	***
P19Aza- - P19Aza+	<0.001	***
SKBR3_4Gy,1.5h - CRL2522_4Gy,1.5h	0.184	n.s.

Table S3. *p*-values (ANOVA-test) for comparison of the amount of heterochromatin of the nuclei.

Comparison	*p*-Value	Significance
CRL2522run - U87	0.454	n.s.
CRL2522run - SKBR3_0Gy	<0.001	***
CRL2522run - P19Aza- .	<0.001	***
U87 - SKBR3_0Gy	<0.001	***
U87 - P19 Aza-	<0.001	***
SKBR3_0Gy - P19 Aza-	<0.001	***
CRL2522run - CRL2522dorm	0.006	**
CRL2522run - CRL2522wake	0.191	n.s.
CRL2522dorm - CRL2522wake	0.042	*
CRL2522run - CRL2522_2Gy,1.5h	<0.001	***
CRL2522run - CRL2522_4Gy,1.5h .	0.690	n.s.
CRL2522_2Gy,1.5h - CRL2522_4Gy,1.5h	<0.001	***
CRL2522_run - CRL2522_2Gy,8h	0.473	n.s.
CRL2522_2Gy,1.5h - CRL2522_2Gy,8h	<0.001	***
SKBR3_0Gy - SKBR3_4Gy,1.5h	0.031	*
P19Aza- - P19Aza+	0.847	n.s.
SKBR3_4Gy,1.5h - CRL2522_4Gy,1.5h	0.002	**

Table S4: *p*-values (ANOVA-test) for comparison of the amount of cohesin of the nuclei

Comparison	*p*-Value	Significance
CRL2522run - U87	0.590	n.s.
CRL2522run - SKBR3_0Gy	0.016	*
CRL2522run - P19Aza- .	0.674	n.s.
U87 - SKBR3_0Gy	0.054	n.s.
U87 - P19 Aza-	0.964	n.s.
SKBR3_0Gy - P19 Aza-	0.001	**
CRL2522run - CRL2522dorm	0.020	*
CRL2522run - CRL2522wake	0.086	n.s.
CRL2522dorm - CRL2522wake	0.167	n.s.
CRL2522run - CRL2522_2Gy,1.5h	0.011	*
CRL2522run - CRL2522_4Gy,1.5h .	<0.001	***
CRL2522_2Gy,1.5h - CRL2522_4Gy,1.5h	0.003	**
CRL2522_run - CRL2522_2Gy,8h	0.002	**
CRL2522_2Gy,1.5h - CRL2522_2Gy,8h	0.177	n.s.
SKBR3_0Gy - SKBR3_4Gy,1.5h	0.020	*
P19Aza- - P19Aza+	<0.001	***
SKBR3_4Gy,1.5h - CRL2522_4Gy,1.5h	<0.001	***

Table S5: *p*-values (ANOVA-test) for comparison of the amount of heterochromatin granula of the nuclei

Comparison	*p*-Value	Significance
CRL2522run - U87	0.013	*
CRL2522run - SKBR3_0Gy	0.940	n.s.
CRL2522run - P19Aza- .	0.776	n.s.
U87 - SKBR3_0Gy	0.004	**
U87 - P19 Aza-	0.028	*
SKBR3_0Gy - P19 Aza-	0.495	n.s.
CRL2522run - CRL2522dorm	0.265	n.s.
CRL2522run - CRL2522wake	0.410	n.s.
CRL2522dorm - CRL2522wake	0.891	n.s.
CRL2522run - CRL2522_4Gy,1.5h .	0.106	n.s.
CRL2522_run - CRL2522_2Gy,8h	0.251	n.s.
SKBR3_0Gy - SKBR3_4Gy,1.5h	0.442	n.s.
P19Aza- - P19Aza+	0,290	n.s.
SKBR3_4Gy,1.5h - CRL2522_4Gy,1.5h	0.149	n.s.

Table S6: *p*-values (ANOVA-test) for comparison of the mean area of heterochromatin granula of the nuclei

Comparison	*p*-Value	Significance
CRL2522run - U87	0.221	n.s.
CRL2522run - SKBR3_0Gy	0.029	*
CRL2522run - P19Aza- .	0.588	n.s.
U87 - SKBR3_0Gy	0.328	n.s.
U87 - P19 Aza-	0.112	n.s.
SKBR3_0Gy - P19 Aza-	0.029	*
CRL2522run - CRL2522dorm	0.504	n.s.
CRL2522run - CRL2522wake	1.000	n.s.
CRL2522dorm - CRL2522wake	0.627	n.s.
CRL2522run - CRL2522_4Gy,1.5h .	<0.001	***
CRL2522_run - CRL2522_2Gy,8h	0.589	n.s.
SKBR3_0Gy - SKBR3_4Gy,1.5h	0.108	n.s.
P19Aza- - P19Aza+	0.003	**
SKBR3_4Gy,1.5h - CRL2522_4Gy,1.5h	0.041	*

Table S7: *p*-values (ANOVA-test) for comparison of the mean intensity of heterochromatin granula of the nuclei

Comparison	*p*-Value	Significance
CRL2522run - U87	0.817	n.s.
CRL2522run - SKBR3_0Gy	0.013	*
CRL2522run - P19Aza- .	0.025	*
U87 - SKBR3_0Gy	0.682	n.s.
U87 - P19 Aza-	<0.001	***
SKBR3_0Gy - P19 Aza-	<0.001	***
CRL2522run - CRL2522dorm	0.412	n.s.
CRL2522run - CRL2522wake	0.844	n.s.
CRL2522dorm - CRL2522wake	0.545	n.s.
CRL2522run - CRL2522_4Gy,1.5h .	<0.001	***
CRL2522_run - CRL2522_2Gy,8h	0.817	n.s.
SKBR3_0Gy - SKBR3_4Gy,1.5h	0.050	n.s.
P19Aza- - P19Aza+	0.012	*
SKBR3_4Gy,1.5h - CRL2522_4Gy,1.5h	0.010	*

Table S8: *p*-values (ANOVA-test) for comparison of the amount of cohesin granula of the nuclei

Comparison	*p*-Value	Significance
CRL2522run - U87	0.871	n.s.
CRL2522run - SKBR3_0Gy	<0.001	***
CRL2522run - P19Aza- .	0.234	n.s.
U87 - SKBR3_0Gy	<0.001	***
U87 - P19 Aza-	0.064	n.s.
SKBR3_0Gy - P19 Aza-	<0.001	***
CRL2522run - CRL2522dorm	0.193	*
CRL2522run - CRL2522wake	0.198	n.s.
CRL2522dorm - CRL2522wake	0.004	**
CRL2522run - CRL2522_2Gy,1.5h	<0.001	***
CRL2522run - CRL2522_4Gy,1.5h .	<0.001	***
CRL2522_2Gy,1.5h - CRL2522_4Gy,1.5h	0.046	*
CRL2522_run - CRL2522_2Gy,8h	<0.001	***
CRL2522_2Gy,1.5h - CRL2522_2Gy,8h	0.075	n.s.
SKBR3_0Gy - SKBR3_4Gy,1.5h	<0.001	***
P19Aza- - P19Aza+	0.026	*
SKBR3_4Gy,1.5h - CRL2522_4Gy,1.5h	0.871	n.s.

Table S9: *p*-values (ANOVA-test) for comparison of the mean area of cohesin granula of the nuclei

Comparison	*p*-Value	Significance
CRL2522run - U87	0.729	n.s.
CRL2522run - SKBR3_0Gy	<0.001	***
CRL2522run - P19Aza- .	0.070	n.s.
U87 - SKBR3_0Gy	0.015	*
U87 - P19 Aza-	0.155	n.s.
SKBR3_0Gy - P19 Aza-	<0.001	***
CRL2522run - CRL2522dorm	0.456	n.s.
CRL2522run - CRL2522wake	0.054	n.s.
CRL2522dorm - CRL2522wake	0.242	n.s.
CRL2522run - CRL2522_2Gy,1.5h	0.376	n.s.
CRL2522run - CRL2522_4Gy,1.5h .	<0.001	***
CRL2522_2Gy,1.5h - CRL2522_4Gy,1.5h	0.002	**
CRL2522_run - CRL2522_2Gy,8h	0.013	*
CRL2522_2Gy,1.5h - CRL2522_2Gy,8h	0.071	n.s.
SKBR3_0Gy - SKBR3_4Gy,1.5h	0.010	**
P19Aza- - P19Aza+	0.656	n.s.
SKBR3_4Gy,1.5h - CRL2522_4Gy,1.5h	0.034	*

Table S10: *p*-values (ANOVA-test) for comparison of the mean intensity of cohestin granula of the nuclei

Comparison	*p*-Value	Significance
CRL2522run - U87	0.876	n.s.
CRL2522run - SKBR3_0Gy	<0.001	***
CRL2522run - P19Aza- .	<0.001	***
U87 - SKBR3_0Gy	0.011	*
U87 - P19 Aza-	0.048	*
SKBR3_0Gy - P19 Aza-	<0.001	***
CRL2522run - CRL2522dorm	0.873	n.s.
CRL2522run - CRL2522wake	0.426	n.s.
CRL2522dorm - CRL2522wake	0.471	n.s.
CRL2522run - CRL2522_2Gy,1.5h	0.072	n.s.
CRL2522run - CRL2522_4Gy,1.5h .	0.004	**
CRL2522_2Gy,1.5h - CRL2522_4Gy,1.5h	0.201	n.s.
CRL2522_run - CRL2522_2Gy,8h	1.000	n.s.
CRL2522_2Gy,1.5h - CRL2522_2Gy,8h	0.120	n.s.
SKBR3_0Gy - SKBR3_4Gy,1.5h	0.254	n.s.
P19Aza- - P19Aza+	0.592	n.s.
SKBR3_4Gy,1.5h - CRL2522_4Gy,1.5h	0.225	n.s.
